# PulseNet: Entering the Age of Next-Generation Sequencing

**DOI:** 10.1089/fpd.2019.2634

**Published:** 2019-07-09

**Authors:** Efrain M. Ribot, Molly Freeman, Kelley B. Hise, Peter Gerner-Smidt

**Affiliations:** Centers for Disease Control and Prevention, Atlanta, Georgia.

**Keywords:** PulseNet, national network, foodborne illness, whole genome sequencing, outbreak detection

## Abstract

Since 1996, PulseNet has served as the national laboratory-based surveillance system for the rapid detection of outbreaks caused by foodborne bacterial pathogens in the United States. For the past two decades, pulsed-field gel electrophoresis was the gold standard subtyping method for the pathogens tracked by PulseNet. A new gold standard is now being implemented with the introduction of cost-effective whole genome sequencing (WGS) for analysis of all the organisms tracked by PulseNet. This transformation is a major undertaking that touches every functional aspect of PulseNet, including laboratory workflows, data storage, analysis management and data interpretation, and language used to communicate information (sequence profile nomenclature system). The benefits of implementing WGS go beyond improved discrimination and precision of the data; it provides an opportunity to determine strain characteristics typically obtained through resource-intensive traditional methodologies, for example, species identification, serotyping, virulence, and antimicrobial resistance profiling, all of which can be consolidated into a single WGS workflow. Such a strategy represents a major shift in the workflows currently practiced in most public health laboratories, but one that brings opportunities for streamlining surveillance activities for the network as a whole. In this study, we provide a brief summary of PulseNet's evolution the past decade along with a general description of the challenges and opportunities that lie ahead.

## Introduction

PulseNet is the nation's molecular subtyping network for foodborne disease surveillance. Using pulsed-field gel electrophoresis (PFGE) as its primary subtyping method, PulseNet has, for over 20 years, facilitated the detection and investigation of outbreaks caused by foodborne bacterial pathogens in the United States (Swaminathan *et al.*, 2001; Gerner-Smidt *et al.*, 2006). While the mission of PulseNet has remained steady, the network has continued to improve the efficiency with which molecular subtyping data are generated, analyzed, and communicated to epidemiologists and other public health partners. It is estimated that the network, by performing real-time PFGE-based laboratory surveillance, prevents an estimated 270,000 cases of foodborne bacterial illnesses and saves the country more than US$500 million in medical costs and loss of productivity annually (Scharff *et al.*, 2016). With an operating cost of approximately US$10–15 million a year, PulseNet continues to provide a great return on investment.

**FIG. 1.**
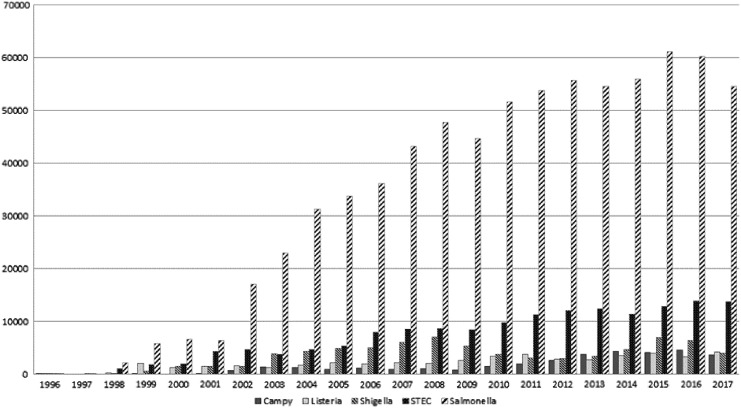
Total submission of PFGE profiles to the PulseNet national database by organisms and year (1996–2017). PFGE, pulsed-field gel electrophoresis.

The network subtypes over 75,000 isolates annually (Fig. 1) (Ribot and Hise, 2016). One of the keys to the success of PulseNet is that subtyping data, along with the accompanying metadata, are archived in a national database and accessible to all network participants on demand. Information is shared with local and national epidemiologists and appropriate public health officials in real-time. Another key feature of PulseNet is the use of the same subtyping and analytical methods by all participants in the network, enabling rapid and efficient data sharing and comparison.

While the core purpose of PulseNet has not significantly changed the past two decades, recent technological advancements in laboratory techniques and availability of powerful analytic tools are having a profound impact on the functionality of the network. The emergence of affordable next-generation sequencing (NGS) technologies in recent years made whole genome sequencing (WGS) a viable and cost-effective subtyping approach for network members. WGS has, along with the development of sophisticated bioinformatics analytical tools, dramatically increased the discriminatory power for subtyping over the previous gold standard, PFGE.

These cutting-edge tools provide new opportunities for laboratories, and the network as a whole, to increase their efficiency by allowing for streamlining and potential consolidation of surveillance and reference workflows since reference characteristics, for example, species identification, serotype, virulence profile, and antimicrobial resistance profile, and more, may be extracted and predicted from the sequence data. However, the adoption of WGS as the new subtyping gold standard has generated a series of new challenges for PulseNet, including determining which analytical approaches to use and how to transfer, store, manage, and analyze the massive amounts of data generated while maintaining the level of quality needed for surveillance activities in a network of more than 80 laboratories.

## Network Organization and Governance

PulseNet USA is a decentralized, collaborative network, which comprises the state and local public health laboratories (PHLs), the Centers for Disease Control and Prevention (CDC), and federal food regulatory agency laboratories (the U.S. Department of Agriculture's Food Safety Inspection Service [USDA-FSIS], Agricultural Research Service [USDA-ARS], Agricultural Marketing Service [USDA-AMS], the Food and Drug Administration's Center for Food Safety Applied Nutrition [FDA-CFSAN], Office of Regulatory Affairs [FDA-ORA], and Center for Veterinary Medicine [FDA-CVM]). PulseNet Central at CDC and the Association of Public Health Laboratories (APHL) coordinate the activities in the network.

**FIG. 2.**
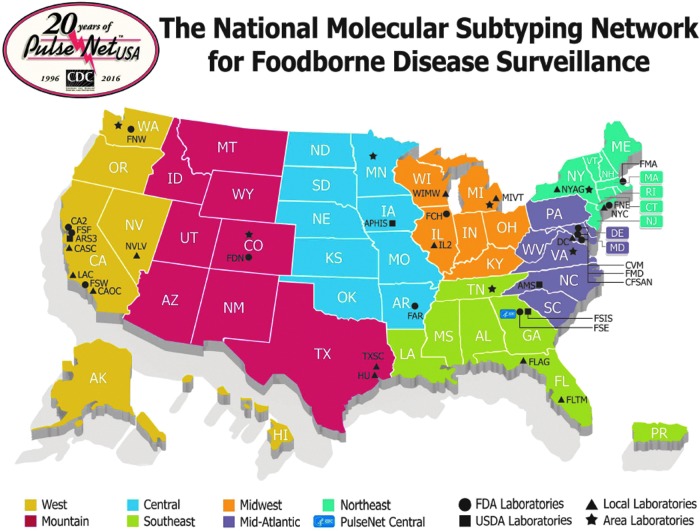
Map showing the geographical organization of PulseNet.

The network is divided into seven areas (Fig. 2) to increase its efficiency and to promote regional collaboration. A designated state laboratory in each area has the responsibility to assist other laboratories in their area with training, troubleshooting laboratory issues, and providing surge capacity for subtyping in case a sudden need arises. Decisions influencing the overall functionality of the network, including the adoption of new technologies and changes in the network's goals and priorities are determined by the PulseNet Steering Committee, which comprises representatives from each member organization and area laboratories participating in the network.

The overall structure and governance of PulseNet has not changed significantly in the past decade, although areas were reorganized in 2015 (Fig. 2). This change was driven by two main goals: (1) achieve a better balanced population distribution across regions and (2) ensure the selected PulseNet Area Laboratories had the capacity to fully support the laboratories in their region with the transition from PFGE to WGS.

## PulseNet Procedures, Data Analysis, and Communication

### Laboratory and analytical methods

Since its beginning, PulseNet relied on PFGE as the primary molecular tool for subtyping because of its epidemiologic relevance, high discriminatory power, and the platform's technical stability (Swaminathan *et al.*, 2001; Gerner-Smidt *et al.*, 2006). Until recently, PulseNet relied on standardized PFGE protocols for subtyping of O157 and non-O157 STEC, *Salmonella enterica*, and *Shigella* spp. (Ribot *et al.*, 2006; Pichel *et al.*, 2012), *Listeria monocytogenes* (Halpin *et al.*, 2010), *Campylobacter* spp. (Ribot *et al.*, 2001), *Vibrio cholera* (Cooper *et al.*, 2006), *Vibrio parahaemolyticus* (Parsons *et al.*, 2007; Kam *et al.*, 2008), and *Cronobacter sakazakii* (Brengi *et al.*, 2012).

In contrast to PFGE, WGS is organism agnostic, that is, the same method is used to sequence all organisms, whereas the analysis of the sequence data differs by organism. WGS has now replaced PFGE as the primary subtyping method for all organisms in PulseNet. However, PulseNet Central will maintain PFGE capacity in the foreseeable future to ensure that national and international laboratories that may lag behind with implementing WGS will receive assistance if needed.

For years, the network closely followed the emergence and evolution of bench top NGS technologies although they were not initially cost effective for implementation in PulseNet. NGS technologies were plagued by sequence quality and error issues; in addition, the rapidly evolving platforms and chemistries were not conducive to the level of standardization needed for successful implementation in PulseNet. In spite of these issues, PulseNet began evaluating NGS devices following its successful use in the 2010 Haiti cholera outbreak (Reimer *et al.*, 2011). It became clear that this technology was key to improving laboratory-based surveillance and likely to overcome many of the limitations of PFGE, including lack of discriminatory power when analyzing common and clonal strains.

When PulseNet evaluated which analytical tools to use for WGS, a number of requirements were considered: (1) the same tools should be used by all participants, ideally harmonized with the international PulseNet community to ensure compatibility of the output between laboratories with no need to go back to the raw sequence data to compare data generated in different laboratories; (2) tools should be user-friendly, push-button tools to ensure that the existing network participants would be able to use them, including interpreting the output, with limited training; (3) the need for local high-capacity computing should be minimized; (4) the data extracted should be in a format that could be stored locally and in the national PulseNet databases along with extensive metadata to facilitate easy analysis; and finally (5) the tools should support an unambiguous, hierarchical nomenclatural system that will enable an assessment of the similarity of sequences of any two isolates to facilitate communication about outbreak related isolates.

The preferred subtyping tools are seven housekeeping gene multilocus sequence typing (MLST), core genome and whole genome MLST (cg/wgMLST), since these are the only ones that fulfill all the requirements mentioned above. The cgMLST schemes used by PulseNet are based on existing publically available schemas to avoid duplication, reduce the workload during validation and implementation, and follow international consensus. The ones adopted by PulseNet include: the *L. monocytogenes* scheme developed by Institut Pasteur, France (http://bigsdb.pasteur.fr/listeria), the *Campylobacter* scheme from the University of Oxford, United Kingdom (https://pubmlst.org/campylobacter), and the *Salmonella* and *Escherichia coli* schemes from the University of Warwick, United Kingdom (https://enterobase.warwick.ac.uk).

The tools used for species and subspecies identification were developed *in-house* at CDC based on average nucleotide identity (CDC, unpublished), whereas tools for serotyping *E. coli* and *Salmonella*, virulence profiling of *E. coli*, antimicrobial resistance, and plasmid profiling were adopted from the tools published on the Center for Genomic Epidemiology (CGE) website (http://genomicepidemiology.org). All the tools have been adapted to run on the server/client database software, BioNumerics v7 (Applied Maths, Sint-Martens-Latem, Belgium), which has been used by PulseNet to analyze and store PFGE and associated metadata since it was introduced ∼20 years ago. The advantage of this software is that all PulseNet participants are familiar with it and will need minimal training to learn to use it with WGS data.

WGS is a rapidly evolving technology from both the device and sequencing chemistries' perspective. For that reason, PulseNet laboratories will likely see an increase in the amount of time and resources that must be dedicated to the revalidation of improved WGS protocols and devices. This will be necessary to ensure that the data used in every laboratory are of the highest quality and level of uniformity possible in addition to insure its compliance with local quality management system and or appropriate accreditation/regulation programs (e.g., CLIA, CAP, ISO, etc.).

### National databases and communication

The adoption of WGS as the new subtyping standard for PulseNet posed immediate challenges to the network's data management, analysis infrastructure, and workflow. For example, a practical solution needed to be found for storing the massive amounts of data generated by sequencing. It was quickly recognized that PulseNet Central at CDC did not have the necessary infrastructure or capacity to handle the amount of data expected to be uploaded annually (∼70,000 isolates) in the context of a traditional national database architecture.

The practical solution to this problem was to store the raw sequence data in the sequence read archive (SRA), a structured, stable, and expandable system for archiving sequence data at the National Center for Biotechnology Information (NCBI) at the US National Institutes of Health (NIH). PulseNet has used FDA's GenomeTrakr database at the SRA since it became available (Stevens *et al.*, 2017) to ensure its data would be easily accessible alongside sequences of foodborne pathogens from other sources. In that respect, the sequence data generated and submitted by PulseNet laboratories to SRA are copied in the European Nucleotide Archive (ENA) and the DNA Data Bank of Japan (DDBJ).

Using the GenomeTrakr database at SRA as the primary repository allowed PulseNet to transform its data management and access business model from closed system, where only member laboratories can access the national database to one where the raw WGS data can be accessed at any time by the general public (non-PulseNet entities). This approach has already led to more open and efficient access of PulseNet WGS data in addition to laying the foundation for the globalization of surveillance of the pathogens tracked by PulseNet.

Data extracted from the raw sequences (MLST and reference characteristics) and extended metadata, which is critical for efficient outbreak surveillance, are still housed in the closed national PulseNet database at CDC that is only accessible to the network participants.

## PulseNet International

**FIG. 3.**
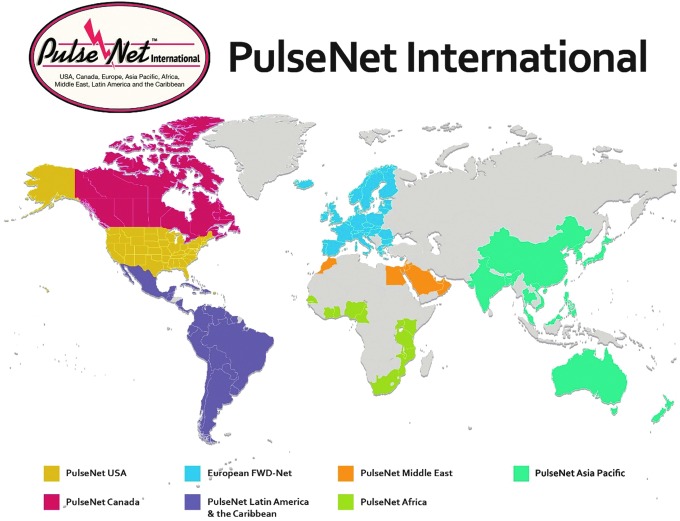
Members of PulseNet International.

PulseNet International is the international umbrella network of national and regional PulseNet networks (Swaminathan *et al.*, 2006). The participants in PulseNet International represent laboratories from more than 80 countries in seven regional networks: PulseNet Africa, PulseNet Asia Pacific, PulseNet Canada, the European FWD (Food and Waterborne Diseases)-Net, PulseNet Latin America and the Caribbean, PulseNet Middle East, and PulseNet USA (Fig. 3). Each network is under the leadership of a coordinating laboratory.

The mission and governance of the network has remained essentially unchanged since the network was established. Its main mission is to facilitate the early detection and investigation of national and international clusters of foodborne infections that may represent outbreaks through laboratory surveillance. When an outbreak is detected, the coordinating laboratory alerts all networks using a web-based communication platform; the PulseNet International forum hosted by PulseNet Canada. Outbreak alerts are often posted in this forum by network participants experiencing a suspected outbreak event or by being in the region with the suspected source of the outbreak.

The governing body of the network is the PulseNet International Steering Committee consisting of the coordinators of the regional networks and chaired by the leader of PulseNet USA. A website officer, who is responsible for the daily maintenance of the website of the network (www.pulsenetinternational.org), is also a member of the Steering Committee. The website contains information about the individual member networks, contact information, information about ongoing activities, presentations, protocols and the preferred hardware and software configurations used by the network. The primary subtyping method in PulseNet International has been PFGE, but like PulseNet USA, the other PulseNet regions are beginning to implement WGS.

A white paper on the implementation of WGS in all PulseNet regions was recently published (Nadon *et al.*, 2017). In that paper, considerations for analytical method selection were described along with the approach chosen to implement the method throughout the system without losing members that currently do not have the capacity to implement it. Learning from early implementers, building WGS capacity and helping those who do not have it are important components of that strategy.

## Present and Future Challenges

The increasing adoption of culture-independent diagnostic tests (CIDTs), for example, PCR panels and enzyme immune assays in the clinical setting for the diagnosis of foodborne illnesses pose short- and long-term challenges to laboratory systems like PulseNet, which uses cultured isolates for their surveillance (Cronquist *et al.*, 2012). However, CIDTs allow for fast identification of the potential cause of illness at the point of care which benefits physicians and patients because treatment can be started much sooner than if the diagnosis was reliant upon culture-based methods.

A short-term solution to the loss of cultures through the adoption of CIDTs is to perform reflex culture of CIDT-positive specimens to obtain an isolate for surveillance. This is typically performed by the PHLs and has resulted in a dramatic increase in their workload. This shift has placed a tremendous pressure on the PHL staff and already limited resources. Given that adoption of CIDTs in the clinical setting is expected to accelerate, it is likely that fewer isolates will be available to PHLs and the CDC for confirmation and subtyping. If this happens, the network's ability to detect and investigate dispersed foodborne disease outbreaks will be severely compromised.

PulseNet is working with public partners and CIDT device manufacturers to identify ways to minimize the negative impacts of CIDT on public health surveillance by (1) educating them about the impact this type of test is having on public health; (2) engaging in conversations and providing suggestions on how to ensure pathogen survival after the specimen is processed; (3) investing in the development of molecular tools for the identification and subtyping of pathogens based on known markers directly from stool and other complex matrices without culture; and (4) developing metagenomic approaches that will allow the recovery and analysis (i.e., sequencing) of genetic material directly from stool and other complex samples with no need for culture. Creating public health solutions to the CIDT challenge that do not depend on the availability of cultures is addressed in more detail in another article in this issue (Carleton *et al.*).

## Discussion and Conclusions

It took 8 years after PulseNet's first experience with WGS in the Haiti cholera outbreak and 5 years following the implementation of WGS as a supplement to PFGE before the technology was implemented in the United States for all the major foodborne pathogens. Which subtyping methods and analytical pipelines to use had to be agreed upon by PulseNet's international partners if the data were to be useful for global surveillance. The pipelines were tested on small scale and then customized into the BioNumerics format, the only software that combines database and analytical capacities with proven track record in a large network of laboratories. Next, the pipelines were validated on a large scale and the informatics infrastructure to handle the data established at CDC. This would not have been possible without support from CDC's Advanced Molecular Detection initiative designed to introduce genomics in public health diagnostics and surveillance, which began in 2013.

At the same time, the end users had to learn how to use and interpret the data generated in actual outbreak scenarios in real-time. In that respect, the PulseNet collaboration with its federal partners at USDA and FDA, including FDAs GenomeTrakr project, proved critical. Establishing sequencing capacity in all states and training the participants was essential. This was achieved through major funding starting in 2016 as a project in the Combating Antimicrobial Resistance in Bacteria (CARB) initiative aimed at determining resistance determinants in all *Salmonella* in support of the National Antimicrobial Resistance Monitoring System (NARMS) (Karp *et al.*, 2017). Even though it initially was not thought of as a PulseNet project, it was quickly realized that PulseNet could use the sequences generated in that project for reference characterization and outbreak surveillance. This is an excellent example of creative and efficient use of federal appropriations.

The implementation of WGS required changes to existing strategies on how these data are stored, managed, and accessed by PulseNet laboratorians compared with PFGE. The strategy of depositing all WGS data generated through the GenomeTrakr database at NCBI solved a critical storage capacity issue for PulseNet, in addition to creating an opportunity for open access of the data by non-PulseNet entities. We hope this will lead to true globalization of foodborne disease surveillance of the pathogens tracked by PulseNet. A major public health outcome of this transformational project is faster and more efficient national and international surveillance, better connectivity with the global public health community, and a safer food supply.

We anticipate that the evolution of PulseNet will continue past the implementation of WGS as the new characterization and subtyping standard, for example, metagenomics, emerges in the United States and abroad. Current and future challenges will continue to reshape the way PulseNet functions from the technical and functional points of view to address emerging food safety challenges.
